# Irisin promotes C2C12 myoblast proliferation via ERK-dependent CCL7 upregulation

**DOI:** 10.1371/journal.pone.0222559

**Published:** 2019-09-13

**Authors:** Jangho Lee, Joon Park, Young Ho Kim, Nam Hyouck Lee, Kyung-Mo Song

**Affiliations:** 1 Research Division of Food Functionality, Korea Food Research Institute, Iseo-myeon, Wanju-gun, Jeollabuk-do, Republic of Korea; 2 Department of Food Biotechnology, Korea University of Science and Technology, Daejeon, Republic of Korea; 3 Research Division of Strategic Food Technology, Korea Food Research Institute, Iseo-myeon, Wanju-gun, Jeollabuk-do, Republic of Korea; Tohoku University, JAPAN

## Abstract

Irisin is an exercise-induced myokine that has various physiological functions, such as roles in energy expenditure, glucose/lipid metabolism, and muscle development. In muscle development, myoblast proliferation is known to be a first step, and recent studies have reported that an increased irisin level is involved in the promotion of cell proliferation in various cell types, including myoblasts. However, the exact mechanism of action by which irisin promotes myoblast proliferation has not been reported. In this study, we aimed to determine the pro-proliferative effect of irisin on C2C12 myoblasts and its mechanism of action. Irisin induced C2C12 cell proliferation and upregulated the mRNA levels of markers of proliferation *Pcna*, *Mki67*, and *Mcm2*. Irisin increased extracellular signal-regulated kinase (ERK) phosphorylation, and U0126, an ERK pathway inhibitor, suppressed irisin-induced C2C12 cell proliferation. Transcriptomic and qRT-PCR analysis showed that *Ccl2*, *Ccl7*, *Ccl8*, and *C3* are potential downstream regulators of ERK signaling that promote C2C12 cell proliferation. Knockdown of *Ccl7* revealed that irisin upregulates chemokine (C-C motif) ligand 7 (CCL7) and subsequently promotes C2C12 cell proliferation. These results suggest that irisin promotes C2C12 myoblast proliferation via ERK-dependent CCL7 upregulation and may aid in understanding how irisin contributes to muscle development.

## Introduction

Irisin is a recently identified myokine that is induced by exercise and stimulates brown-fat-like development of white fat and energy expenditure in humans and mice [1, 2]. Boström *et al*. [2] found that transgenic mice overexpressing peroxisome proliferator-activated receptor-γ co-activator 1α (PGC1α), a well-known mediator of the effect of exercise, exhibit upregulation of fibronectin type III domain-containing protein 5 (FNDC5) expression, and irisin is released by proteolytic cleavage of FNDC5. Irisin is mainly secreted from muscle and has been detected in various organs, such as the adipose tissue, brain, liver, kidney, and muscle itself [1, 3].

Irisin has physiological functions, such as roles in glucose/lipid metabolism, the regulation of cell proliferation, endothelial function, mitochondrial biogenesis, and the inhibition of oxidative stress, in various organs. Several studies have shown that irisin promotes the proliferation of endothelial cells [4] and osteoblasts [5]. In skeletal muscle, irisin induces myogenesis and mitochondrial biogenesis and protects against muscle atrophy and bone loss [6], and a recent study has reported that irisin promotes C2C12 cell proliferation [7, 8]. However, the mechanisms of action involved in irisin-induced proliferation of C2C12 myoblasts have not been elucidated.

Chemokine (C-C motif) ligand 7 [CCL7, also known as monocyte-chemotactic protein 3 (MCP3)] is a chemokine that functions as a chemoattractant for leukocytes such as monocytes and neutrophils. CCL7 is widely expressed in various cell types and can contribute to anti-inflammatory responses; however, abnormal CCL7 levels can lead to inflammation and tumorigenesis [9, 10]. Some studies have shown that CCL7 promotes the proliferation of smooth muscle cells [11] and astrocytes [12]; however, a pro-proliferative effect of CCL7 on myoblasts has not been reported so far.

In this study, we aimed to evaluate the pro-proliferative effect of irisin on C2C12 myoblasts and its mechanisms of action. Irisin was found to increase C2C12 cell proliferation through the ERK signaling pathway. Transcriptomic analysis revealed that irisin-regulated genes were mainly involved in the immune response, and *Ccl2*, *Ccl7*, *Ccl8*, and *C3* were upregulated by irisin via the ERK signaling pathway in C2C12 cells. CCL7 was confirmed to promote C2C12 cell proliferation, and knockdown of *Ccl7* abolished irisin-induced C2C12 cell proliferation.

## Materials and methods

### Materials

U0126 (an MEK1/2 inhibitor) and SB203580 (a p38 inhibitor) were purchased from Selleckchem (Houston, TX, USA). Human recombinant irisin (≥90%) was purchased from Caymen Chemical (Ann Arbor, MI, USA). Murine C3 recombinant protein was purchased from Novus (Littleton, CO, USA). Murine MCP-3 (CCL7) recombinant protein was purchased from Peprotech (Rocky Hill, NJ, USA). Antibodies specific to detect Thr202/Tyr204-phospho ERK, total ERK, Tyr180/182-phospho p38, total p38 were obtained from Cell Signaling Biotechnology (Beverly, MA, USA). Antibodies specific to β-actin (C-4) were obtained from Santa Cruz Biotechnology (Santa Cruz, CA, USA). The protein assay kit was obtained from Bio-Rad Laboratories (Hercules, CA, USA).

### Cell culture and MTS assay

Murine myoblast C2C12 cells were maintained in DMEM containing 10% FBS (Gibco, Grand Island, NY, USA), 100 U/ml of penicillin and 100 mg/ml of streptomycin at 37°C in a 5% CO_2_ humidified incubator. To estimate cell viability, C2C12 cells were seeded at 1×10^3^ cells/well in 96-well plates and incubated at 37°C in a 5% CO_2_ incubator. After 24 hours, the C2C12 cells were treated with irisin, ccl7, or c3 recombinant proteins for indicated times, and 100 μL of MTS solution in the presence of phenazine methosulphate was added to each well. After 1 hour of incubation, the absorbance levels for formazan at 490 and 630 nm were measured by using a microplate reader.

### Western blotting

For Western blot assays, C2C12 cells (2×10^5^ cells / dish) were seeded in 10 cm dishes for 24 hours. The cells were serum-starved for 4 hours and then treated with irisin for indicated times or concentrations. Then, the cells were collected and washed twice with cold PBS, before lysis in Cell Lysis Buffer (Cell Signaling, Beverly, MA, USA) and maintained on ice for 30 min. The lysate protein was washed via centrifugation and the concentration determined using a DC Protein Assay kit (Bio-Rad Laboratories) following manufacturer’s instructions. The lysate was subjected to 10% sodium dodecyl sulfate–polyacrylamide gel electrophoresis (SDS-PAGE) and transferred to a polyvinylidene difluoride (PVDF) membrane (Millipore, Immobilon^®^-P transfer membrane). After transferring, the membranes were incubated with the specific primary antibodies at 4°C overnight. Protein bands were visualized using a chemiluminescence detection kit (ATTO, Tokyo, Japan) after hybridization with a horseradish peroxidase (HRP)-conjugated secondary antibody.

### Quantitative real-time RT-PCR

Total RNA was isolated using the RNeasy^®^ Mini Kit (Qiagen, Valencia, CA, USA) according to the manufacturer’s instructions. Reverse transcription of RNA was performed with the ReverTra Ace^®^ qPCR RT Master Mix (Toyobo, Osaka, Japan). First-strand cDNA was prepared from 1 μg total RNA. The real-time PCR reaction was performed in a volume of 20 μl containing 0.1 μg of cDNA, 1 μM of each primer (Table 1), and Power SYBR^®^ Green PCR Master Mix (Applied Biosystems, Carlsbad, CA). The thermal cycling was carried out in a StepOnePlus Real-Time PCR system (Applied Biosystems) with a program of 95°C for 5 min., followed by 40 cycles with denaturation at 95°C for 5 sec., annealing and elongation at 60°C for 10 sec. The gene expression levels were normalized to the expression level of the GAPDH housekeeping gene. Relative gene expression changes, calculated using the 2^-ΔΔCT^ method, are reported as number-fold changes compared to those in the control samples. For microarray analysis, total RNA was pooled from n = 3 biological replicates and processed in BioCore (Seoul, Republic of Korea) as described below.

**Table 1:** Primer sequences for qRT-PCR.

Species	Gene	Primer sequence	
		Forward	Reverse
Mouse	*ccl2*	AGCACCAGCACCAGCCAACT	CAGGTGACTGGGGCATTGAT
	*ccl7*	GCTGCTTTCAGCATCCAAGTG	CCAGGGACACCGACTACTG
	*ccl8*	TCTACGCAGTGCTTCTTTGCC	AAGGGGGATCTTCAGCTTTAGTA
	*c3*	CCAGCTCCCCATTAGCTCTG	GCACTTGCCTCTTTAGGAAGTC
	*Pdgfra*	TCCATGCTAGACTCAGAAGTCA	TCCCGGTGGACACAATTTTTC

### Microarray for gene expression analysis

The quality and quantity of total RNAs were assessed by Agilent bioanalyzer 2100 analysis. Analysis of gene expression was performed using GeneChip^®^ Mouse Gene 2.0 ST Arrays (Affymetrix, Santa Clara, CA, USA). *In situ* synthesis of eleven pairs of oligonucleotide probes was conducted for each gene. Single stranded-DNA (ssDNA) was fragmented and labeled from 500 ng of total RNA (GeneChip^®^ WT PLUS Reagent Kit Manual, Affymetrix). After DNA fragmentation, ssDNA was subjected to hybridization for 16 hours at 45°C (60 rpm; GeneChip^®^ Mouse Gene 2.0 ST Array). The GeneChips were washed stained, and then scanned using the Affymetrix GeneChip Scanner 3000 7G. The scanned data were converted to intensity values, which were normalized and log transformed.

### Knockdown of CCL7

For knockdown of CCL7, C2C12 cells were transfected with scrambled (Cat No. SN-1002, Bioneer, Daejeon, Republic of Korea) or 10 μM murine CCL7 siRNA (Cat No. 1336008 (#1), 1336007 (#2), Bioneer, Daejeon, Republic of Korea) by using Lipofectamine^®^ RNAiMAX (Invitrogen, Carlsbad, CA, USA), following the manufacturer’s suggested protocols. The transfected cells were then used in subsequent experiments.

### Statistical analysis

Where appropriate, data are expressed as the means ± standard deviation (SD), and significant differences were determined using one-way ANOVA (Analysis of Variance) followed by Tukey’s post-hoc test. A probability value of *p* < 0.05 was used as the criterion for statistical significance.

## Results

### Irisin promotes proliferation of C2C12 myoblasts

**Fig 1:**
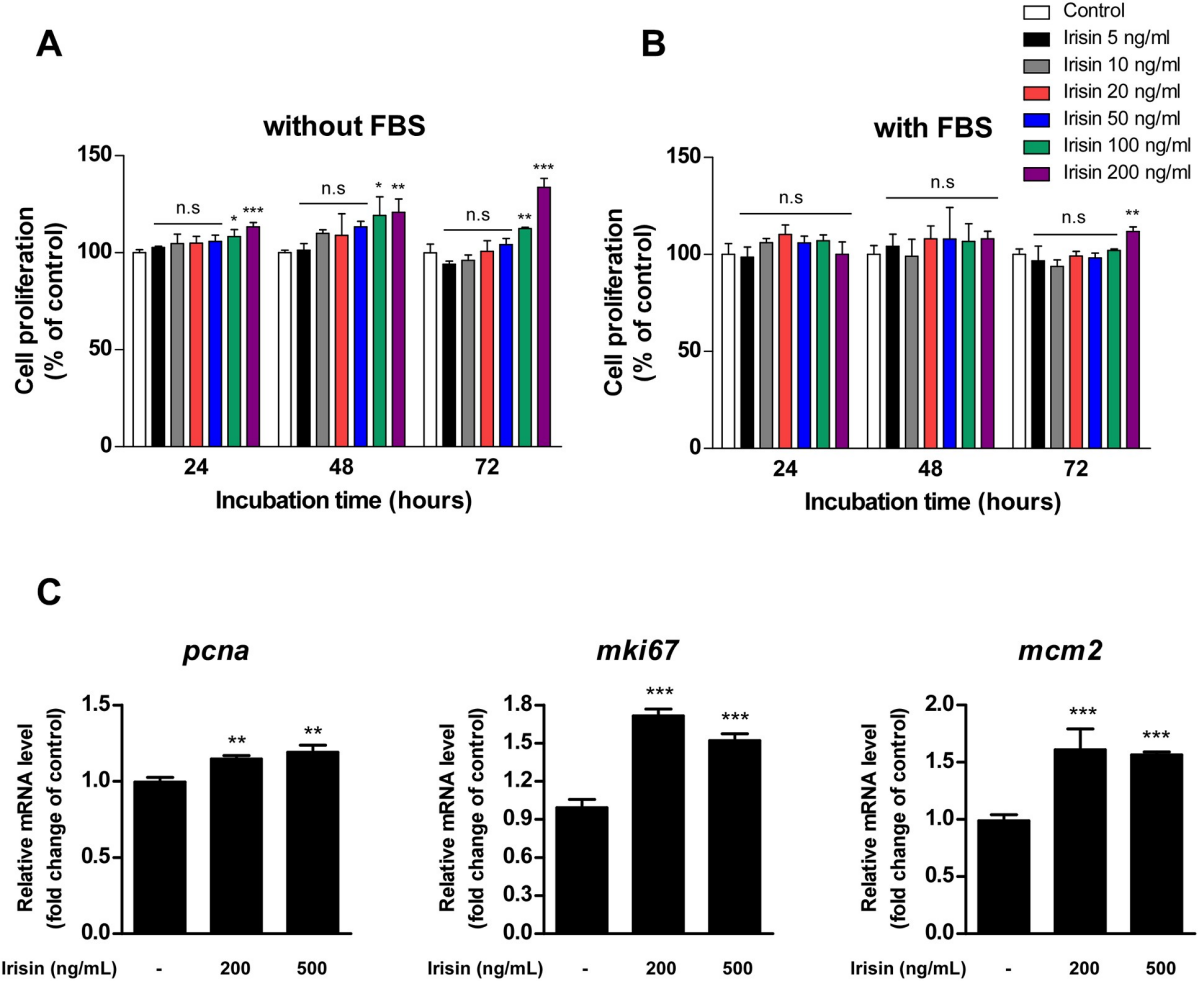
Irisin promotes C2C12 cell proliferation. **(A, B)** C2C12 cells were treated with irisin for indiciated times in culture medium **(A)** without FBS or **(B)** with FBS. Cell proliferation (%) was measured by MTS assay. Data are represented as the means ± SD (n = 3). Asterisk symbols (*, **, and ***) indicate significant differences between control group and irisin-treated groups (*p* < 0.05, *p* < 0.01, and 0.001, respectively). n.s, not significant (*p* > 0.05). **(C)** Irisin upregulates gene expression of the cell proliferation markers, *pnca*, *mki67*, and *mcm2* in C2C12 cells. C2C12 cells were treated with 200 or 500 ng/mL of irisin for 24 hours. Relative mRNA levels were measured by qRT-PCR. Bar graph indicates mean ± SD (n = 3). Asterisk symbols (** and ***) indicate significant differences between control group and irisin-treated groups (*p* < 0.01 and 0.001, respectively).

The effect of irisin treatment on C2C12 myoblast proliferation was evaluated. Irisin treatment significantly promoted C2C12 cell proliferation in a time- and dose-dependent manner (Fig 1A). In addition, this effect was attenuated in the presence of fetal bovine serum (FBS) (Fig 1B). Proliferating cell nuclear antigen (PCNA), a marker of proliferation Ki-67 (MKI67), and minichromosome maintenance complex component 2 (MCM2) are known to be representative markers of cell proliferation [13]. Irisin significantly increased the mRNA expression of the genes encoding PCNA (*Pcna*), Ki-67 (*Mki67*), and MCM2 (*Mcm2*) in C2C12 cells (Fig 1C).

### Irisin promotes C2C12 cell proliferation mainly through activation of ERK signaling

**Fig 2:**
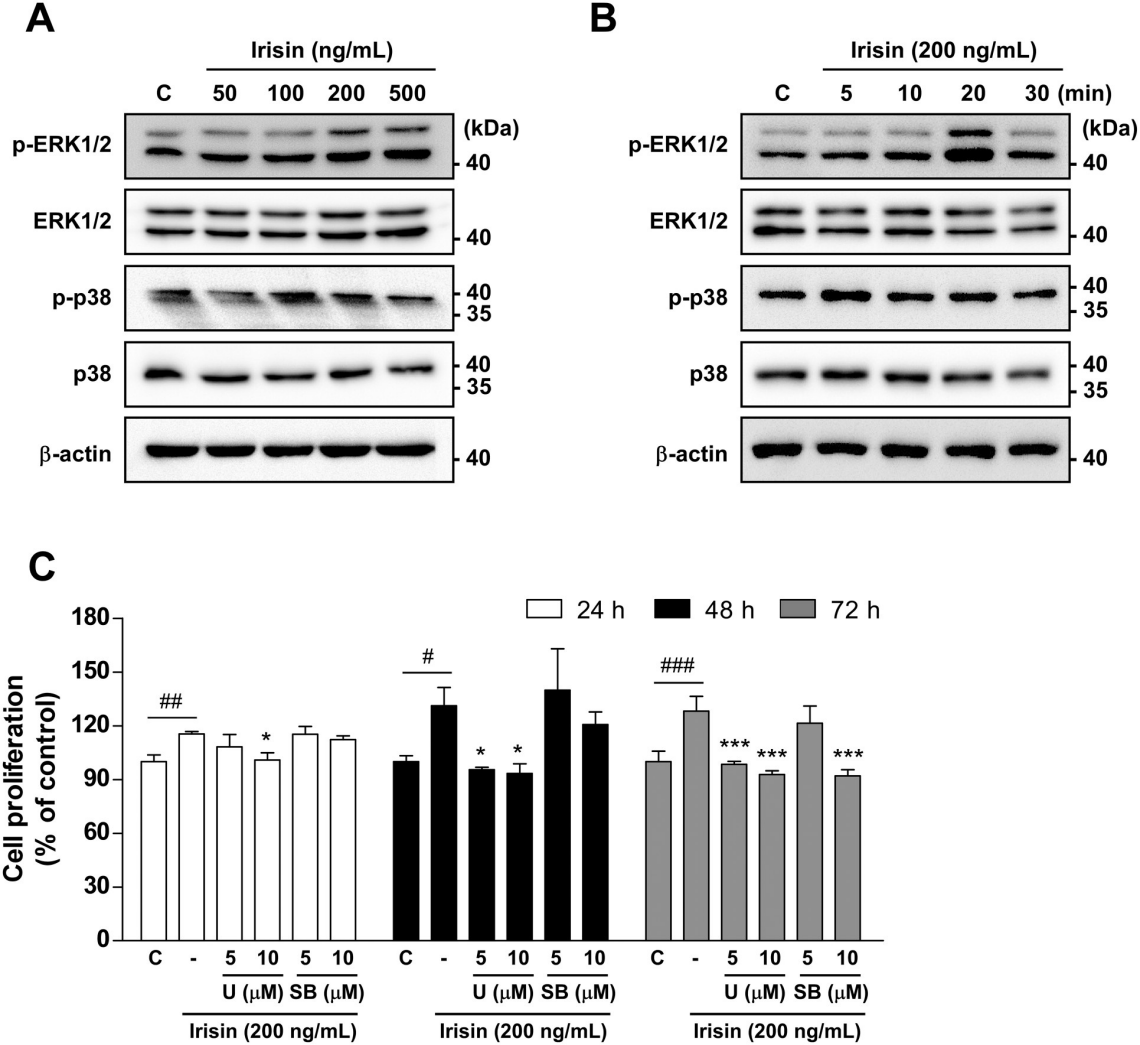
Irisin promotes C2C12 cell proliferation via activation of ERK. **(A, B)** Irisin phosphorylated ERK1/2 in a **(A)** dose-dependent and **(B)** time-dependent manner. Expression and phosphorylation levels of indicated proteins were determined by Western blotting. **(C)** Irisin-induced C2C12 cell proliferation was sensitive to inhibition of the ERK signaling pathway. Cell proliferation (%) was measured by MTS assay. Data are represented as the means ± SD (n = 3). Hash symbols (#, ##, and ###) indicate significant differences between control and irisin-treated groups (*p* < 0.05, 0.01, and 0.001, respectively) and asterisk symbols indicate significant differences between the irisin-treated group and the irisin and inhibitor co-treated groups (*p < 0.05 and ***p < 0.001). U, U0126 (an MEK1/2 inhibitor); SB, SB203580 (a p38 inhibitor).

The ERK and p38 signaling pathways have been reported to be involved in irisin-mediated promotion of proliferation of various cell types [4, 14, 15]. To assess the upstream signaling pathways involved in irisin-promoted proliferation of C2C12 cells, the effects of irisin treatment on the phosphorylation of ERK and p38 were evaluated. Irisin increased ERK but not p38 phosphorylation in a dose-dependent manner in C2C12 cells (Fig 2A). Irisin increased ERK phosphorylation levels the most at 20 min after treatment, while p38 phosphorylation levels were not changed by irisin treatment (Fig 2B). To confirm the effects of ERK and p38 pathway inhibition on irisin-promoted C2C12 cell proliferation, C2C12 cells were treated with an ERK pathway inhibitor, U0126, or a p38 pathway inhibitor, SB203580, in the presence of irisin. U0126 treatment strongly suppressed irisin-promoted C2C12 cell proliferation at 24, 48, and 72 hours, however, SB203580 inhibited C2C12 cell proliferation only at 72 hours after irisin treatment (Fig 2C).

### Irisin upregulates the immune-related transcriptome in C2C12 cells

**Fig 3:**
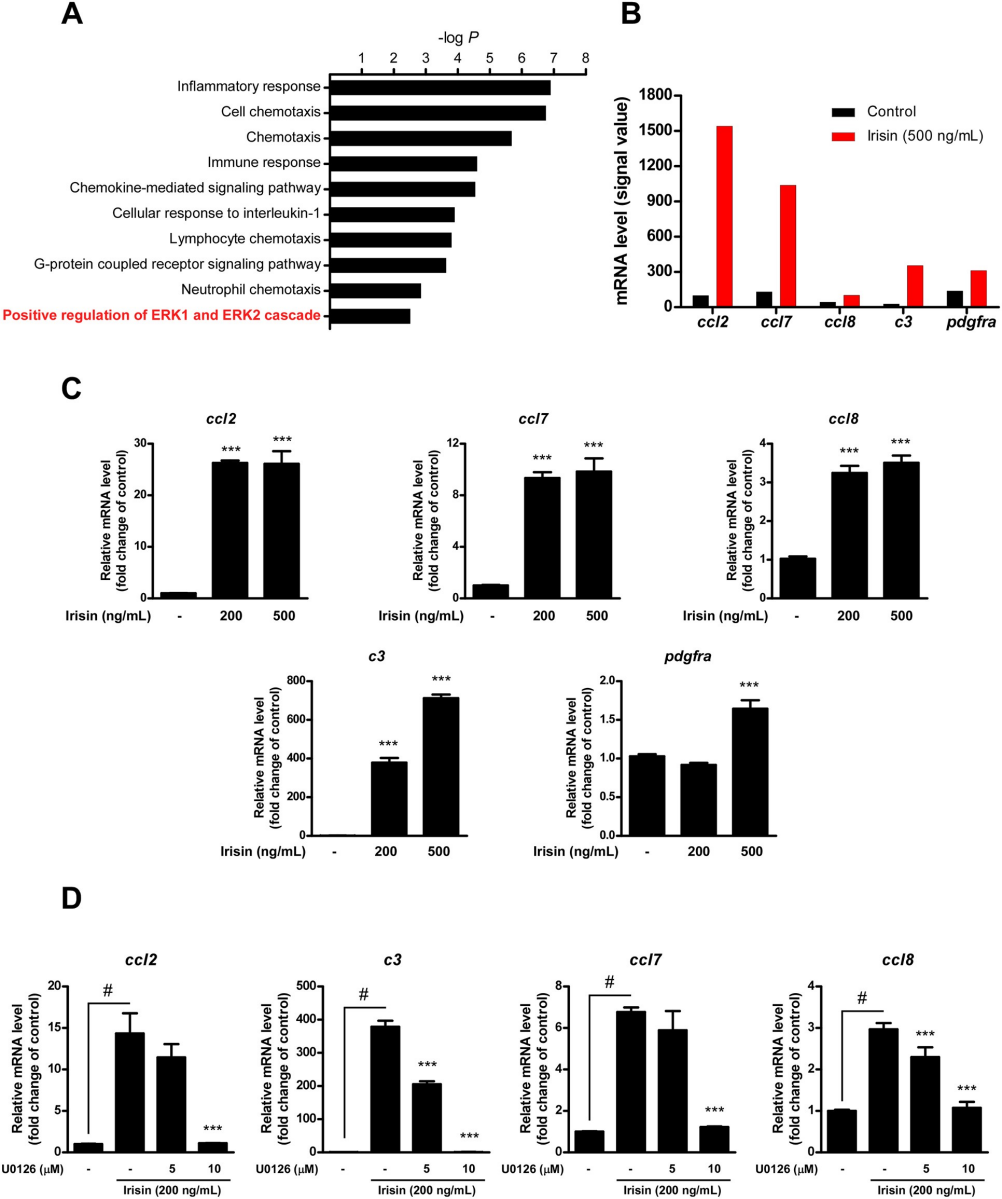
Transcriptomic analysis of irisin-regulated gene expression in C2C12 cells. **(A)** Gene ontology (GO) analysis of irisin-regulated transcriptome in C2C12 cells. The graph shows–log *p* values (modified Fisher’s exact *P*-values) obtained from GO analysis in terms of ‘biological processes’ using microarray data [fold change ≥ 2, pooled samples (n = 3) per group]. **(B)** mRNA expression levels of enriched genes involved in positive regulation of ERK signaling pathway obtained from microarray data. **(C)** Validation of expression levels of genes involved in positive regulation of ERK cascade (*Ccl2*, *Ccl7*, *Ccl8*, *C3*, and *Pdgfa*). Relative mRNA levels were analyzed by qRT-PCR using SYBR Green dye (n = 3 per group).

To evaluate which genes are involved in irisin-promoted C2C12 cell proliferation, we performed transcriptomic analysis using a microarray. A total of 57 genes were upregulated (fold change ≥ 2) and 18 genes were downregulated (fold change ≤ 0.5) following irisin treatment (500 ng/ml) compared to levels in the control group (S1 Fig). These 75 genes were subjected to gene enrichment analysis in terms of biological processes, and immune response-related biological processes (inflammatory response, chemotaxis, etc.) were largely associated with irisin-regulated gene expression changes (Fig 3A). Five genes, *Ccl2*, *Ccl7*, *Ccl8*, *C3*, and *Pdgfra* were involved in both immune response and positive regulation of ERK cascade (Fig 3B and S1 Table). Upregulation of these genes following irisin treatment was validated in C2C12 cells by qRT-PCR analysis (Fig 3C). In addition, treatment with the ERK inhibitor U0126 significantly suppressed irisin-induced *Ccl2*, *C3*, *Ccl7*, and *Ccl8* gene expression in C2C12 cells (Fig 3D).

### CCL7 promotes proliferation of C2C12 myoblasts

**Fig 4:**
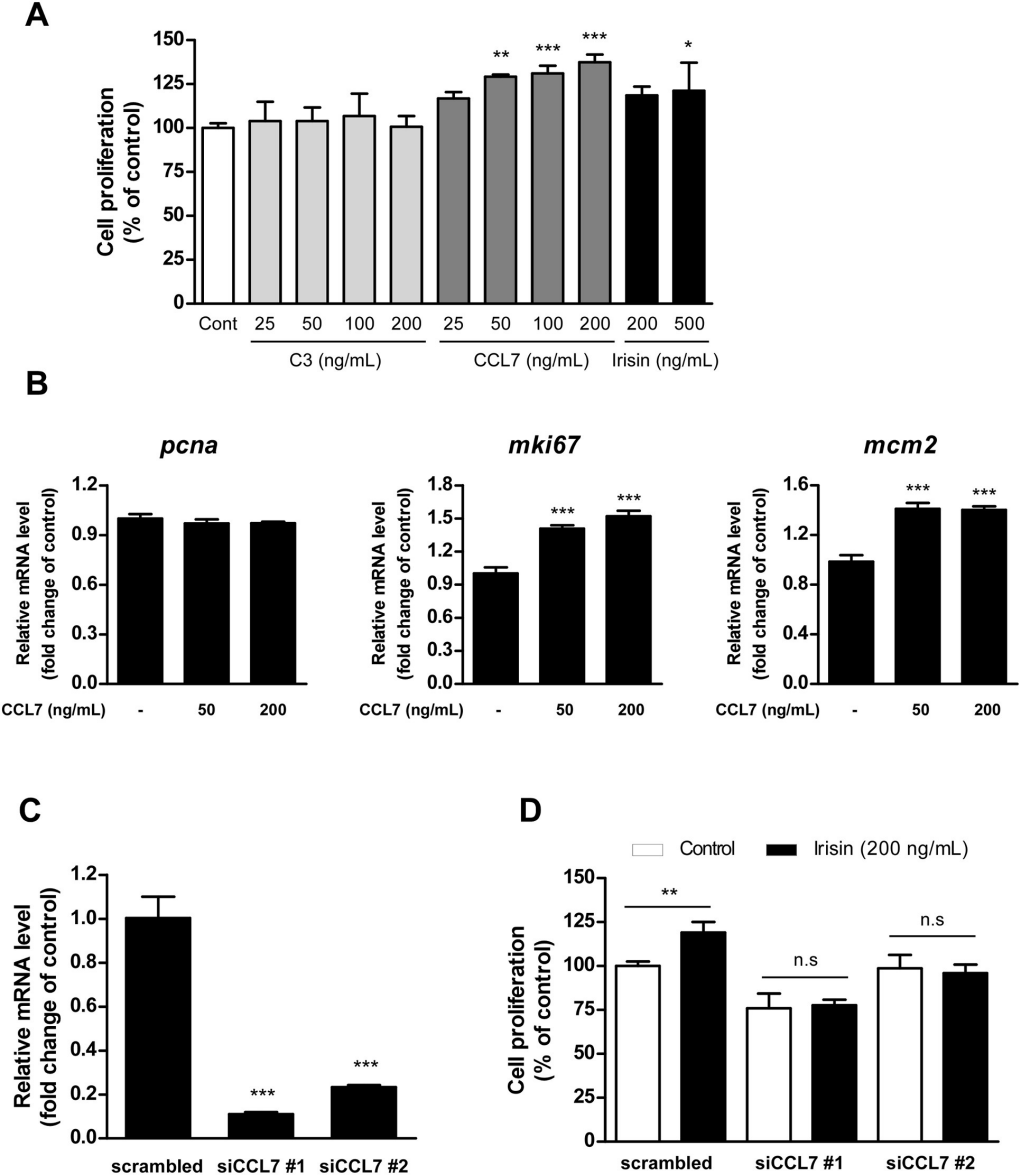
CCL7 promotes C2C12 cell proliferation. **(A)** Treatment with murine CCL7 but not C3 recombinant protein promoted C2C12 cell proliferation. The cells were serum-straved for 4 hours and then treated with C3, CCL7, or irisin for 24 hours. Cell proliferation (%) was measured by MTS assay. Data are represented as the means ± SD (n = 3). Asterisk symbols (*, **, and ***) indicate significant differences between control group and C3, CCL7 or irisin-treated groups (*p* < 0.05, p < 0.01, and 0.001, respectively). **(B)** Knockdown of CCL7 suppresses irisin-induced C2C12 cell proliferation. Cells were transfected with scrambled (negative control) or indicated siRNA for 48 hours with or without treatment of irisin. Cell proliferation (%) was measured by MTS assay. Data are represented as the means ± SD (n = 4). An asterisk symbol (**) indicates significant difference between control group and irisin-treated group (*p* < 0.01) in C2C12 cells transfected with scrambed siRNA. n.s, not significant (*p* < 0.05). **(C)** Knockdown of *Ccl7* diminished *Ccl7* mRNA expression in C2C12 cells. Cells were transfected with scrambled (negative control) or indicated siRNA for 48 hours. Relative mRNA levels were analyzed by qRT-PCR using SYBR Green dye (n = 3 per each group). Data are represented as the means ± SD (n = 4). An asterisk symbol (***) indicates significant difference between negative control group (scrambled) and siCCL7 groups (*p* < 0.001). **(D)** Knockdown of *Ccl7* suppressed gene expression of the cell proliferation markers *Pnca*, *Mki67*, and *Mcm2* in C2C12 cells. The C2C12 cells were transfected with scrambled (negative control) or indicated siRNA for 48 hours, then, the cells were treated with 200 ng/mL of irisin for 24 hours. Relative mRNA levels were measured by qRT-PCR. Bar graph indicates mean ± SD (n = 3). An asterisk symbols (**) indicates significant difference between control group and irisin-treated groups (*p* < 0.01). n.s, not significant (*p* > 0.05).

Several studies have reported that C3 and CCL7 induce proliferation of lung and neuronal cells, respectively [12, 16]. Thus, we evaluated whether C3 and CCL7 promote C2C12 cell proliferation. CCL7 but not C3 significantly increased C2C12 cell proliferation in a dose-dependent manner (Fig 4A). CCL7 also increased expression of the cell proliferation markers *Mki67* and *Mcm2* in C2C12 cells (Fig 4B). To further confirm the effect of CCL7 on C2C12 cell proliferation, we performed *Ccl7* knockdown in C2C12 myoblasts (Fig 4C). While irisin promoted C2C12 cell proliferation, *Ccl7* knockdown suppressed irisin-induced C2C12 cell proliferation (Fig 4D).

## Discussion

The recently identified protein irisin is an exercise-induced myokine involved in the mechanism of the effects of exercise on weight reduction [2], insulin resistance [17], and skeletal muscle development [8]. For muscle development, myoblast proliferation is required for the development of myotubes and muscle fibers [18]. Consistent with our results, some studies have shown that irisin induces C2C12 myoblast proliferation [7, 8]. In addition, it has been reported that irisin promotes osteoblast and endothelial cell proliferation through the ERK and/or p38 MAPK signaling pathways [5, 19]. Thus, we assumed that the ERK and/or p38 MAPK signaling pathways may be associated with irisin-induced proliferation of C2C12 cells, and our results indeed showed that irisin increases ERK phosphorylation and that irisin-induced cell proliferation is inhibited by a specific ERK pathway inhibitor in C2C12 cells. These results suggest that irisin promotes C2C12 myoblast proliferation through the ERK signaling pathway.

To identify which genes are regulated by irisin treatment, we performed transcriptomic analysis in C2C12 myoblasts. Interestingly, most irisin-regulated genes were involved in immune response-related biological functions, and five of these genes (*Ccl2*, *Ccl7*, *Ccl8*, *C3*, and *Pdgfra*) were confirmed to be upregulated via the ERK signaling pathway in C2C12 cells. A previous study has also shown that *Ccl2* and *Ccl7* are upregulated by irisin treatment in C2C12 cells [8]. Treatment with an ERK pathway inhibitor, U0126, inhibited irisin-induced *Ccl2*, *Ccl7*, *Ccl8*, and *C3* gene expression in C2C12 cells, suggesting that irisin upregulates the expression of three chemokines (*Ccl2*, *Ccl7*, and *Ccl8*) and *C3* in C2C12 myoblasts.

According to our results, we hypothesized that *Ccl2*, *Ccl7*, *Ccl8*, and *C3* encode potential downstream regulators of irisin that promote C2C12 cell proliferation. Since C3 and CCL7 are reported to induce proliferation in some cells [12, 16], the effects of C3 and CCL7 on C2C12 cell proliferation were further evaluated. Our results showed that CCL7 but not C3 induced C2C12 cell proliferation. In addition, knockdown of *Ccl7* abolished irisin-induced C2C12 cell proliferation. CCL7 has been reported to show pro-proliferative effects in human vascular muscle cells via ERK1/2 and PI3K signaling pathways [20]. Moreover, interfering with tumor necrosis factor (TNF)-induced CCL7 expression using a tissue factor pathway inhibitor suppressed human vascular muscle cell proliferation [21]. CCL7 has been reported to be abnormally expressed in the lung [22] and renal carcinoma [23], and to show pro-proliferative effect in the colorectal cancer cell line HCT116 [24]. Collectively, these results suggest that CCL7, which is upregulated by irisin treatment, functions as a positive regulator of C2C12 myoblast proliferation.

In conclusion, we have revealed the mechanism of action by which irisin induces C2C12 myoblast cell proliferation. Namely, irisin increases the expression of immune response-related genes such as *Ccl2*, *Ccl7*, *Ccl8*, and *C3* via the ERK signaling pathway. CCL7 was confirmed to promote C2C12 cell proliferation. Collectively, these results demonstrate that irisin promotes C2C12 myoblast proliferation by upregulating CCL7 via the ERK signaling pathway. These results may aid in understanding how irisin regulates the proliferation of myoblasts, the initial step in muscle development.

## Supporting information

S1 Fig Heatmap visualization of differential gene expression levels between control and irisin-treated C2C12 cells. C2C12 cells were treated with irisin (500 ng/mL) for 24 hours for transcriptomic analysis. Green and red colors indicate the signal intensity of gene expression levels.
(TIF)

S1 Table Gene ontology analysis. (DOCX)
