# Supplementary figures and images for: Irisin promotes C2C12 myoblast proliferation via ERK-dependent CCL7 upregulation
Source: PLoS One. 2019 Sep 13;14(9):e0222559. doi: 10.1371/journal.pone.0222559 (PMC6743866; doi:10.1371/journal.pone.0222559)

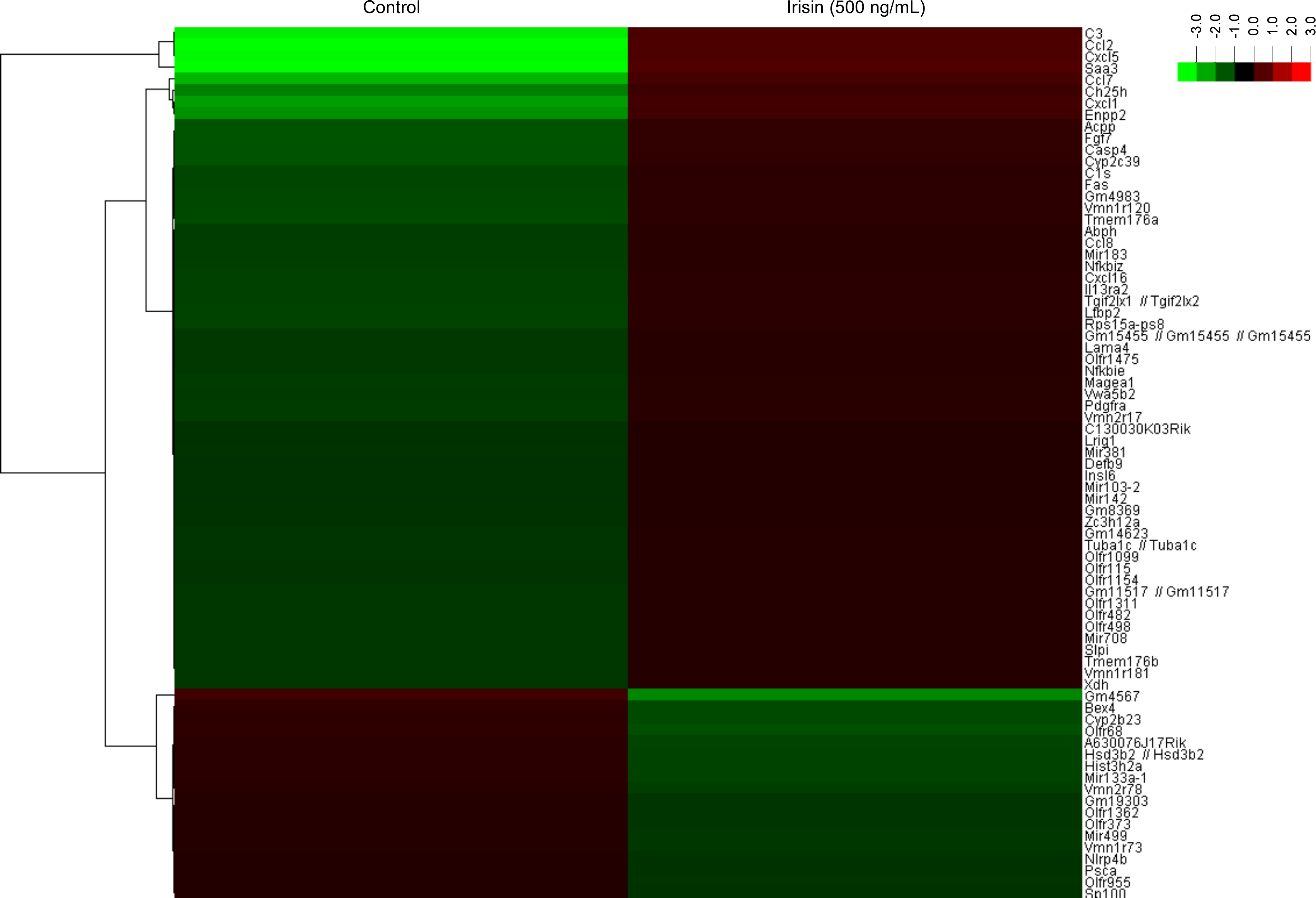

Supplement: S1 Fig — C2C12 cells were treated with irisin (500 ng/mL) for 24 hours for transcriptomic analysis. Green and red colors indicate the signal intensity of gene expression levels. (TIF) [file pone.0222559.s001.tif]
