# Supplementary material for: Irisin promotes C2C12 myoblast proliferation via ERK-dependent CCL7 upregulation
Source: PLoS One. 2019 Sep 13;14(9):e0222559. doi: 10.1371/journal.pone.0222559 (PMC6743866; doi:10.1371/journal.pone.0222559)
Supplement: S1 Table — (DOCX) [file pone.0222559.s002.docx]

**Table 1. Gene ontology analysis**

| **Term** | ***p*-value** | **Genes** |
| --- | --- | --- |
| GO:0006954~inflammatory response | 1.26E-07 | CXCL1, NFKBIZ, CASP4, CCL2, CXCL5, C3, CCL8, ZC3H12A, NLRP4B, FAS, CCL7 |
| GO:0060326~cell chemotaxis | 1.79E-07 | CXCL1, CCL2, CXCL5, ENPP2, PDGFRA, SAA3, CCL8 |
| GO:0006935~chemotaxis | 2.09E-06 | CCL2, CXCL5, ENPP2, CXCL16, PDGFRA, CCL8, CCL7 |
| GO:0006955~immune response | 2.52E-05 | CXCL1, CCL2, CXCL5, ENPP2, CCL8, SLPI, FAS, CCL7 |
| GO:0070098~chemokine-mediated signaling pathway | 2.88E-05 | CXCL1, CCL2, CXCL5, CCL8, CCL7 |
| GO:0071347~cellular response to interleukin-1 | 1.26E-04 | CCL2, SAA3, CCL8, ZC3H12A, CCL7 |
| GO:0048247~lymphocyte chemotaxis | 1.60E-04 | CCL2, CXCL16, CCL8, CCL7 |
| GO:0007186~G-protein coupled receptor signaling pathway | 2.34E-04 | CXCL1, OLFR1362, CCL2, CXCL5, CCL8, OLFR115, OLFR1099, CCL7, OLFR1311, OLFR498, OLFR1154, OLFR68, OLFR373, OLFR1475, OLFR955, OLFR482 |
| GO:0030593~neutrophil chemotaxis | 0.0014121 | CXCL1, CCL2, CCL8, CCL7 |
| GO:0070374~positive regulation of ERK1 and ERK2 cascade | 0.0030997 | CCL2, C3, PDGFRA, CCL8, CCL7 |
| GO:0007608~sensory perception of smell | 0.003175 | OLFR1311, OLFR498, OLFR1154, OLFR68, OLFR1362, OLFR1475, OLFR373, OLFR482, OLFR955, OLFR1099, OLFR115 |
| GO:0071222~cellular response to lipopolysaccharide | 0.0045198 | MIR142, CCL2, CXCL16, ZC3H12A, MIR381 |
| GO:0071356~cellular response to tumor necrosis factor | 0.0053015 | CCL2, CCL8, ZC3H12A, CCL7 |
| GO:0002548~monocyte chemotaxis | 0.0072938 | CCL2, CCL8, CCL7 |
| GO:0071230~cellular response to amino acid stimulus | 0.0096902 | MIR708, MIR183, PDGFRA, MIR103-2 |
| GO:2001199~negative regulation of dendritic cell differentiation | 0.0127699 | TMEM176B, TMEM176A |
| GO:2000427~positive regulation of apoptotic cell clearance | 0.0127699 | CCL2, C3 |
| GO:0050729~positive regulation of inflammatory response | 0.0174236 | CCL2, CCL8, CCL7 |
| GO:0071346~cellular response to interferon-gamma | 0.0201171 | CCL2, CCL8, CCL7 |
| GO:0032496~response to lipopolysaccharide | 0.025358 | CXCL1, CXCL5, SLPI, FAS |
| GO:0010884~positive regulation of lipid storage | 0.0316248 | C3, ZC3H12A |
| GO:0048245~eosinophil chemotaxis | 0.0316248 | CCL2, CCL7 |
| GO:0050731~positive regulation of peptidyl-tyrosine phosphorylation | 0.0417082 | FGF7, ENPP2, PDGFRA |
| GO:1900745~positive regulation of p38MAPK cascade | 0.0531752 | XDH, ZC3H12A |
| GO:0010468~regulation of gene expression | 0.0762085 | MIR183, ZC3H12A, FAS, MIR499 |
| GO:0043547~positive regulation of GTPase activity | 0.0798606 | CCL2, CCL8, CCL7 |
| GO:0045765~regulation of angiogenesis | 0.0860914 | SP100, ENPP2 |
| GO:0019373~epoxygenase P450 pathway | 0.0919542 | CYP2B23, CYP2C39 |
| GO:2000379~positive regulation of reactive oxygen species metabolic process | 0.0948717 | XDH, ZC3H12A |
